# Factors Associated with Cardiovascular Disease Risk among Employees at a Portuguese Higher Education Institution

**DOI:** 10.3390/ijerph19020848

**Published:** 2022-01-13

**Authors:** Maria Piedade Brandão, Pedro Sa-Couto, Gonçalo Gomes, Pedro Beça, Juliana Reis

**Affiliations:** 1 School of Health [ESSUA], Agras do Crasto-Campus Universitário de Santiago, University of Aveiro, Edifício 30, 3810-193 Aveiro, Portugal; 2 Center for Health Technology and Services Research [CINTESIS], Campus Universitário de Santiago, University of Aveiro, 3800-193 Aveiro, Portugal; 3 Department of Mathematics [DMAT], University of Aveiro, 3810-193 Aveiro, Portugal; p.sa.couto@ua.pt (P.S.-C.); juliana.reis@ua.pt (J.R.); 4 Center for Research and Development in Mathematics and Applications [CIDMA], University of Aveiro, 3810-193 Aveiro, Portugal; 5 Department of Communication and Art [DECA], University of Aveiro, 3810-193 Aveiro, Portugal; goncalo@ua.pt (G.G.); pedrobeca@ua.pt (P.B.); 6 Research Institute for Design, Media and Culture [ID+], University of Aveiro, 3810-193 Aveiro, Portugal; 7 Digital Media and Interaction [DigiMedia], University of Aveiro, 3810-193 Aveiro, Portugal

**Keywords:** cardiovascular disease risk, workplace settings, central Portugal, health risk factors

## Abstract

This study aimed to estimate the prevalence of risk factors for cardiovascular disease (CVD) and to assess the CVD risk (CVDRisk) in a sample of workers at a specific workplace: a higher education institution in Portugal. Data were collected using a questionnaire (e.cuidHaMUs.QueST^®^) with 345 HEI workers from June 2017–June 2018 with a high response rate (93.3%). Two constructs of risks for CVD were considered: (i) metabolic risk and hypertension (CVDRisk1); and (ii) modifiable behavioural risk (CVDRisk2). Logistic regression analyses were used to establish a relationship between risk indexes/constructs (CVDRisk1 and CVDRisk2) and groups of selected variables. The most prevalent CVD risk factor was hypercholesterolaemia (43.2%). Sixty-eight percent of participants were in the construct CVDRisk1 while almost half of the respondents were in CVDRisk2 (45.2%). The consumption of soft drinks twice a week or more contributed to a significantly increased risk of CVD in CVDRisk1. Lack of regular exercise and lack of daily fruit consumption significantly increased the risk of CVD in CVDRisk2. The challenge to decision makers and the occupational medical community is to incorporate this information into the daily practices of health surveillance with an urgent need for health promotional education campaigns in the workplace.

## 1. Introduction

Cardiovascular diseases (CVD) are the noncommunicable diseases (NCD) with the highest mortality rates worldwide [1]. CVD is estimated to cause 17.9 million deaths each year or 31% of all global deaths. It is one of the main types of NCD [2]. In Portugal, CVD is the main cause of mortality and leads to approximately 35,000 deaths per year [3]. 

CVD is a disorder of the heart and blood vessels and is a consequence of various risk factors; therefore, most cardiovascular diseases can be prevented by addressing those risk factors. These risk factors can be divided into two major groups: metabolic risk factors and modifiable behavioural risk factors [4]. Metabolic risk factors can be divided into four main metabolic changes: increased blood pressure (hypertension), overweight, high blood glucose levels (hyperglycaemia), and high levels of fat in the blood (hyperlipidaemia). Modifiable behavioural risk factors are personal behaviours that have a strong influence on health and increase the risk of NCDs, especially CVD, such as smoking, sedentary lifestyle, unhealthy diets, and alcohol abuse. Therefore, modifiable behavioural risk factors are those that have the most significance because they can be controlled by humans [5,6].

Portugal has the highest level of sedentary lifestyle in the European Union [7]. In addition, there is an increasing tendency towards the prevalence of CVD in developed countries, due to increased salt consumption, reduced physical activity, and lowered levels of health literacy [8]. However, university-staff-based studies have shown that they share very similar risk factors for CVD such as obesity [9], physical inactivity [10,11], and smoking [12]. Recently, there was a study about the prevalence of hypertension in Brazil, which showed that 58.3% of university employees self-reported obesity as the most frequently occurring comorbidity [13]. 

Several observational studies have established that behavioural modifications and alterations to modifiable behavioural risk factors are associated with a reduction in cardiovascular disease mortality [14,15,16,17,18,19]. Furthermore, strategies to improve public health and create environments conducive to behavioural change can facilitate a supportive environment. Based on the scientific evidence, the World Health Organization released guidelines for the assessment and management of cardiovascular risk for the prevention of cardiovascular disease [20].

Scientific evidence has confirmed the relationship between occupation and cardiovascular health [21,22,23]. Consequently, knowledge related to risk factors, including workplace factors, is important to promote successful specific health programs. In Europe, there are limited data on cardiovascular health in working populations [24,25]. The higher education institutions (HEIs) in Europe, which are members of the UK Healthy Universities Network, are gradually introducing health promotion strategies using a “whole university approach”. This strategy aims to develop a supportive ethos and culture to embed health into the university, focusing on the whole population [26,27,28]. 

HEIs can play a critical role in offering and promoting strategies to reduce the risk factors of CVD in students, faculty, and staff. The issue is to know which risk factors are the most important to start with. In Portugal, the real risk factors of NCDs and, more specifically, the risk factors associated with CVD among workers at HEIs are still unknown. Thus, our goal here is to estimate the prevalence of CVD risk factors and to assess the cardiovascular risk in a sample of workers at an HEI in Portugal.

## 2. Materials and Methods

### 2.1. Study Design and Subjects

This study used a cross-sectional design. The study population of the University of Aveiro (UA) is composed of professors, researchers, and technical, administrative, and management staff. The data were collected in the occupational medicine (OM) service of UA from June 2017 to June 2018. All participants were OM users. At the time, the total population of UA was 1722 employees. Under Portuguese law, the employee is required to attend consultations and examinations determined by the doctor specialising in OM. The same law requires periodic examinations to be carried out every two years for most workers and yearly from the age of 50. The integrated health monitoring system in universities (e.cuidHaMUs) program is carried out in the OM area, and all workers are free to participate [29]. The sample size was 322 individuals (18.7% of the workers in the UA population), and the response rate of those invited to participate in our study was 93.3% (workers in OM consultations in UA). 

This study is based on a self-administered questionnaire and participants who went to the occupational medicine consultation were invited to participate. As it was not possible to randomly select people for the occupational medicine consultation (due to logistics issues), we considered the sample to be a convenience one. For a 95% confidence interval, with a total finite population of 1722, with a sample of 322 and an estimated prevalence guess of 50%, the margin of error was 4.93. After the estimation of the prevalence of the cardiovascular risks 1 and 2 of 68.2% and 45.2% (2), the margins of error were 4.59% and 4.90%, respectively.

### 2.2. Data Collection Procedures

UA employees were invited to participate in this study at the waiting room of OM at the university health centre (CSU) during their medical appointment. All participants responded to a short and customised questionnaire: e.cuidHaMUs.QueST^®^. The e.cuidHaMUs.QueST^®^ is a multidimensional assessment tool for working adults. It is a simple instrument that collects information on the self-perception of health, behaviours regarding health, and lifestyles. It allows one to collect some diagnostic results from consultation with OM [29].

The e.cuidHaMUs.QueST^®^ has 52 questions as a checklist across six domains: (1) sociodemographic characteristics (gender, age, civil status, department, and academic degree); (2) current self-rated health (self-perception of general health, smoking habits, weight, and height); (3) current diseases and pain (current diseases, locale, duration, and intensity of pain); (4) daily eating habits (taking breakfast, eating fruits, eating vegetables, drinking soda, eating fast food, and drinking alcohol); (5) physical activity habits (if they practice weekly regular exercise, the average daily soft mobility time, and the average weekly hours of practice of regular exercise); and (6) periodic diagnostic tests (current arterial pressure and annual exams such as uric acid, glucose, cholesterol, spirometry, visual and auditory screening, simple electrocardiogram, erythrogram, and leukogram). Only the related outcome measures with cardiovascular risks (see next section) are presented in this paper. All the questionnaires, with the exception of the last part, were completed by the users while waiting for the consultation in the lounge (it takes four to five minutes to answer). The last part (related to “periodic diagnostic tests”) was fulfilled with the help of the OM doctors. More details about the e.cuidHaMUs.QueST^®^ tool can be read elsewhere [29].

### 2.3. Outcome Measures

The clinical and biological parameters were analysed by considering reference values recommended by the World Health Organization (WHO) for a BMI rating [30] including the new recommendations for blood pressure measurement by the American Heart Association (AHA) [31,32] and the guidelines on diabetes [33] and dyslipidaemias [34].

Two major groups (constructs) of risk for cardiovascular disease were named: (1) cardiovascular risk (CVDRisk1), corresponding to metabolic and hypertension risk factors for NCD; and (2) cardiovascular risk (CVDRisk2), which corresponds to modifiable behavioural risk factors for NCD.

Based on the WHO [1], CVDRisk1 participants should have at least one of four indicators:

1. Hypercholesterolaemia (TC ≥ 190 mg/dL);
2. Hypertension (systolic BP ≥ 140 and/or diastolic BP ≥ 90 mmHg);
3. Diabetes (fasting glucose ≥ 126 mg/dL);
4. Obesity (BMI ≥ 30).

Participants were allocated to medium- or high-risk CVDRisk2 if they had at least one of four indicators:

1. Smoker (current smoking status);
2. Physical inactivity (no physical exercise and soft mobility time spent/day < 15 min);
3. Unhealthy diet (no eating fruits or no eating vegetables or eating fast food twice or more days/week);
4. Harmful use of alcohol (medium or high risk).

The dependent variable was categorised as presence or absence of cardiovascular disease risk and was compared with the other study variables. Total cholesterol (TC) was considered elevated when above 190 mg/dL [34], and diabetes when above 126 mg/dL [33].

Drinking habits were calculated for each type of drink and the frequency of alcohol use (proportioned to 365 days) based on the WHO [35] criteria for risk of alcohol consumption per day. The average amount of pure alcohol [g/day] was calculated to be volume [mL] × Vol-% × 0.793 [g/mL]. Those who drink more than 400 (male) or 280 (female) g/week of pure alcohol are considered heavy drinkers, against 168 (male) or 112 (female) g/week, who are considered moderate drinkers [35].

In European countries, one unit of alcohol is defined as having 8 g of pure ethanol and it is recommended that adults should not drink more than three (or exceptionally four) units of alcohol per day [36]. Our study considered a current drinker to be those who drank more than 25 g/day of pure alcohol.

All biophysical data such as blood pressure, glucose, and total cholesterol were obtained at the OM service and analysed in an accredited laboratory. Height and weight were self-reported by the participants, and the corresponding categorisation for body mass index (BMI) was used [30]: underweight (below 18.5 kg/m^2^), normal weight (between 18.5 and 24.9 kg/m^2^), overweight (between 25 and 29.9 kg/m^2^), or obese (over 30 kg/m^2^). Self-reported measures were considered a good practice because scientific evidence has shown no significant difference in direct (objective) or self-reported measures [37].

### 2.4. Statistical Analysis

Summary statistics were reported as the mean and standard deviations for continuous variables and as counts and percentages for categorical variables. The statistical association between the two cardiovascular risks was assessed by the chi-squared test (χ^2^).

Potential factors were associated with the cardiovascular risk indexes (CVDRisk1 and CVDRisk2) and were explored throughout univariable analysis using a binary logistic regression model. The multivariable analysis was performed only for the significant variables, presenting *p* < 0.05 in the univariable model. The odds ratios (OR) and the correspondent 95% confidence intervals (95% CI) were calculated for both analyses. The multivariable regression model was performed using a forced entry method (all the considered significant variables in the univariate model are entered into the model at the same time). The assumption for the binary logistic regression models (Hosmer and Lemeshow test) was verified. Multicollinearity between the independent variables was addressed [38], but no strong associations/correlations between them could be found. All statistical analyses were performed using SPSS^®^ software, version 22.0 (SPSS, Inc., Chicago, IL, USA), and *p*-values under 0.05 were considered significant.

### 2.5. Ethical Considerations

This study was approved by the Ethics and Deontology Council of the UA under registration number 14/2016. All participants read and signed a free and informed consent form after being informed of the study’s objectives, anonymity, confidentiality, and the voluntary nature of their participation. We also emphasise that all data collected were anonymous.

## 3. Results

Table 1 presents the sociodemographic as well as self-rated health, physical, and eating habits of this sample. More than half of the sample are women (50.2%) between 45 and 64 years old (70.7%), married (69.7%), with a higher-level qualification (83.3%), and working in the natural and technical sciences (51.0%). Most of the responses showed a good self-rated health status (54.5%) and with no chronic pain (74.1%). Almost half of the participants (47.0%) reported excessive fat accumulation (overweight and obesity). Most of the participants have a breakfast everyday (90.9%), eat fruits and vegetables daily (70.8% and 66.8%, respectively), eat fast food once a week (47.6%), never drink soda (49.4%), and drink alcohol at least once a week (61.6%). Most participants do not practice any type of regular physical activity (58.3%) and walk between 15 and 30 min/day or less (69.4%). When programmed physical exercise is reported (41.7%), then time spent is around 1–2 h/week (41.4%).

Regarding metabolic and hypertension risk factors (CVDRisk1), 43.2% of the subjects presented hypercholesterolaemia, 19.6% presented hypertension, 10.1% presented hyperglycaemia, and only 1.3% presented diabetes. For the considered modifiable behavioural factors (CVDRisk2), 17.7% presented physical inactivity, 15.0% were smokers, 14.2% drank more than 25 g of alcohol per day, 13.2% presented an unhealthy diet, and 9.9% were obese (≥30 kg/m^2^).

Table 2 presents a contingency table between CVDRisk1 and CVDRisk2. Concerning cardiovascular risk, the majority of participants were found in the CVDRisk1 group (68.2%) and almost half of the participants were in the CVDRisk2 group (45.2%). In addition, 32.5% of the participants presented both cardiovascular risks while only 19.2% presented none of the risks. However, no statistical association could be found between the two cardiovascular risks, indicating the measurement of two different constructs.

Table 3 presents the factors associated with CVDRisk1 and CVDRisk2 from univariable and multivariable logistic regression results. The univariable analysis shows the factors associated with CVDRisk1: being a worker in the organic units related to courses in social and human sciences (OR = 4.982; 95% IC = 1.418–17.509), those who do not exercise regularly (OR = 1862; 95% IC = 1.131–3.065), and those who do not eat fruit every day (OR = 2.252; 95% IC = 1.251–4.054). Exercising on a regular daily basis and eating fruit every day were also confirmed as risk factors for CVDRisk1 in the multivariate results.

Significant factors associated with CVDRisk2 in the univariable analysis included being male (OR = 1.894; 95% IC = 1.157–3.100), drinking soft drinks once a week (OR = 2.312; 95% IC = 1.318–3.715), or twice a week or more (OR = 3.429; 95% IC = 1.818–6.467). Only drinking soft drinks once per week (OR = 2.410; 95% IC = 1.353–4.291) or twice per week or more (OR = 3.387; 95% IC = 1.874–7.651) was confirmed to be a risk factor in the multivariable model.

## 4. Discussion

This preliminary study offers new information to the medical community and decision makers on risk factors associated with DCV in a population of HEI workers. To the best of our knowledge, this is a pioneering study in Portugal because it provides information on the cardiovascular risk of workers in HEIs; it is the first study using the e.cuidHaMUs program [29].

Monitoring health to implement countermeasures is the foundation of this study [29,39]. A major issue in this observational study is the collection of reliable data, which explains why the periodic OM consultations seem to be the ideal time and place for collection. People are more easily accessible (almost unhurried) when they are waiting for their medical appointment; thus, they can answer the proposed questionnaire. Second, the objective data (blood tests) in OM are obligatorily performed for the appointment (e.g., blood glucose and cholesterol). Third, the doctor in service can help the participants to complete the questionnaires.

One quarter of the participants reported chronic illness, and the majority (54.5%) perceived themselves to be in good health, which is higher than the national average (46.4%), although it remained beneath the European Union (EU) average (20.2%) [40]. This could be related to the educational level of most participants: a higher level of education contributes to greater perceptions of health [40,41]. However, the World Health Organization states that Portuguese are more pessimistic about their state of health due to the country’s socioeconomic status [40].

This study shows that most participants (69.5%) do not walk over 30 m/day as recommended (World Health Organization, 2016, 2018), and more than half of the participants (58.3%) do not practice any type of regular physical activity. Nearly one-third of this population walks less than 15 min a day and about a fifth has sedentary behaviour. The results concur with the Portuguese scenario regarding sedentary lifestyles. Studies carried out by the National Health Service revealed that 72% of Portuguese adults “never” or “rarely” engage in exercise or sport, and only 23% complied with the WHO recommendations. Physical activity is critical for proper heart function. Exercise provides oxygen and nutrients to tissues and helps the cardiovascular system to function more efficiently [42,43]. Therefore, it is not surprising that cardiovascular diseases have a negative impact on the life of sedentary people [44,45]. Several studies have established the existence of a relationship between lack of physical exercise and the development of metabolic risk factors (diabetes, hypercholesterolaemia, hypertension, and obesity) [45,46,47,48]. The literature has shown that exercising in its various forms (sport or leisure, or even replacing the car with a bicycle or walking) contributes substantially to the reduction of obesity [49], smoking habits [50], and, mainly, lowering cardiovascular risk [51]. Although the workers that were evaluated presented parameters similar to those of the national context, they still need monitoring to avoid a potential increase in the inactivity prevalence with a consequent increase in cardiovascular risks.

Nearly half of the participants presented hypercholesterolaemia (43.2%; a biological NCD and CVD risk factor), and about one-fifth (19.6%) presented hypertension. Thus, it is not surprising that among UA workers, almost three quarters are at metabolic cardiovascular risk (along with hypertension). These factors are very well-documented and they contribute to the development of cardiovascular disease [2]; hypertension is the biggest contributor of all risk factors [52].

Regarding the population in question, 68.2% of the population has CVDRisk1, i.e., these participants have metabolic or hypertension risk factors. Of this, 45.2% is associated with CVDRisk2, which is a modifiable behavioural risk factor. One-third of the participants (32.5%) are in both types of risk categories, and only 19.2% are not associated with any of the risk factor groups, revealing the growing need to raise awareness of cardiovascular disease risk factors [53,54]. Modifiable risk factors are the most significant because they can be manipulated or controlled by humans. In this study, these would be risk factors aimed at prevention or improvement.

We used the WHO concepts to construct two cardiovascular risk groups [1]; therefore, the designation “modifiable risk factors” refers to those factors that the reader can very easily consider to be susceptible to immediate management. We do not mean that the other factors (metabolic risk factors and hypertension) are not also subject to modification [55]. However, this can be achieved in a more indirect way and therefore is not so easily understood by the common reader. The non-modifiable risk factors studied here were gender and age. Male gender was the only metric significantly associated with CVDRisk2 (OR = 1.984; 95% CI = 1.157–3.100). Smoking, physical inactivity, unhealthy diet, and harmful use of alcohol are well known as lifestyle risk factors which are related to cardiovascular diseases with greater probability of risk of premature death due to NCDs in males rather than females in all WHO regions [1].

This study confirms that certain factors such as a diet low in fruit and vegetables and lack of exercise are associated with the development of cardiovascular risk—in this case, CVDRisk1. These factors are directly and indirectly associated with increased body weight [52,56]. Given that fat consumption in Europe has increased over the past two decades, and noting that nearly half of the study sample (46.3%) is overweight (including obesity) and is at risk for CVD, this result suggests that the dietetics factors among all behavioural risk factors are the biggest contributor to CVD risk [52]. Of all the behavioural risk factors, dietary factors are the biggest contributors to CVD mortality risk across Europe [30,52]. The results of this study may show a trend in the prevalence of overweight among workers at the targeted HEI. Measures to control and reduce body weight should be adopted. Occupational medicine, as an integral part of health support services in HEI, may play a very important role in helping to implement measures not only for individual cases but also for the HEI community as a whole. Promoting regular exercise and nutritional counselling to prevent the development of overweight/obesity could be the initial steps to reach those goals.

Drinking soft drinks even once a week is a predictor of cardiovascular risk; however, here the association is with CVDRisk2. Regardless of the type of cardiovascular risk in question, we are faced with three factors that can be modified individually. However, people alone cannot always change their behaviour, and interventions are usually complex [57,58].

We compared our results with those of similar studies carried out worldwide in King Faisal University, University of Jos, Taibah University, and University of Brasilia [12,59,60,61]; the prevalence of hypercholesterolaemia was considerably higher in our study than in Taibah University (43,2% vs. 23.4%), while the prevalence of hypertension was lower than in the King Faisal University, University of Jos, Taibah University, and University of Brasilia studies (19.6% vs. 22.1%, 48.5%, 52.7%, and 27.5%, respectively). The same studies found higher prevalence of smoking and sedentarism than in our population. National data from 2019 show that the prevalence of hypertension reaches 26.4% in the Portuguese, an increase of 1.1 percentage points since 2014 [62]. Such data allow us to suppose that the sample of workers targeted in this study has lower blood pressure levels when compared to the national reality, as well as similar realities (HEIs) in other parts of the world. Our results may have been influenced by the population’s higher level of education when compared to the national context and, therefore, higher levels of self-care regarding health. However, when compared with similar contexts, we can reflect on the possible contribution of the national health system and the improvement of prevention services developed in recent years. Currently, Portugal has a lower preventable mortality rate than the European Union average (140 deaths vs. 161 deaths, respectively, per 100,000 population in 2016) [63]. These facts should not be a reason for passivity, but should promote efforts to eliminate these risk factors to come closer to the Northern European countries (especially Scandinavian countries) [7].

Institutions are increasingly implementing strategies to promote health in the workplace. They concentrate on prevention while compliance with health and safety practices is neglected [64]. One of the risk factors most alarmingly associated with work is sedentary lifestyle because most jobs have a fixed schedule associated with a specific location. With advanced age, this tendency towards sedentary habits increases, particularly when associated with the time each individual spends sitting per day [65,66,67]. In this study, we deal with an aging work population (70.7% are aged between 45 and 64 years). As we know, aging is the condition that is most related to the appearance of cardiovascular problems. Thus, we emphasise that the natural aging process, in relation to the evaluated workers, recommends the adoption of preventive measures, such as increasing daily exercise, improving nutrition, and improving lifestyles to mitigate the usual increase in cardiovascular risks.

In recent decades, some strategies to improve nutrition and increase physical activity, as proposed by passive organisations, have been followed and even replicated [68,69]. However, the operationalisation of processes for monitoring the health status of workers such as the one presented here (e.cuidHaMUs) can encourage decision makers’ willingness to apply more appropriate and focused strategies, considering the results presented here.

The current study does have some limitations, such as its observational cross-sectional design, its small and non-randomised sample, and the fact that results only partially reflect the perceived health status and determinants of CVD in a population of Portuguese HEI workers. Despite using convenience sampling, the results seem to reflect the reality of UA [70]: female gender and the level of higher education were higher (50.2% vs. 54.3% and 83.3% vs. 87.6%, respectively). Furthermore, our sample is similar to that presented in another study of CVD [71,72]. Although the response rate was high (93.3%), only 322 participants were recruited here in this study, or 18.7% of the total UA workers. The difficulties in recruiting more workers were simply based on our staffing limitations. However, the sample under study is larger when compared to other studies with a broader focus on health problems in HEIs, as can be observed in the Saudi Arabia, Spain, and Brazil studies (322 participants vs. 233, 124, and 201, respectively) [9,10,73].

The strength of this study is that it includes two risk factors groups: a group that includes modifiable behavioural risk factors and a group that includes metabolic risk factors and hypertension. When analysed independently, these contribute to more than half of the sample and are more likely to develop cardiovascular disease. This propensity was observed in about a third of the participants regardless of the constructs analysed.

Remembering what the Ottawa letter in 1996 said, that health is built in places where people *learn*, *work*, *play*, *and love* each other, the question remains: what is the role of HEIs in the health of individuals and the community? In this study, we show the health status of workers in a Portuguese HEI so that the results can contribute to a deep reflection on the part of those responsible for managing and guiding campus life and its workers. In general, workers remain for around 40 years in the same institution and take with them behaviours and attitudes (good or bad) acquired over that time. If there are initiatives capable of creating projects and actions aimed at the continuous improvement of the relationship between risk factors for CVD and the health of workers in the workplace, we will certainly have healthier aging and, especially, a better quality of life due to the reduction of complications associated with cardiovascular diseases.

Future work will increase the sample size and continue to monitor the population. We will seek information from other HEIs and prospectively analyse the risk of developing CVD. We will also evaluate the impact of this current study on Portuguese HEI occupational health policies. Exploration of the hereditary, clinical, and pathological factors that contribute to the current proportions of people at risk for CVD is another interesting perspective to include in future research.

## 5. Conclusions

The metabolic (and hypertension) risk factors as well as modifiable behaviours to CVD contribute to more than half of Portuguese HEIs workers’ risk of cardiovascular disease when analysed independently. This propensity was observed in about a third of the participants regardless of the constructs analysed. The results confirm the urgent need to invest in research regarding HEI workplaces.

## Figures and Tables

**Table 1:** Participants’ sociodemographic characteristics, health self-rated, physical habits, and eating habits.

Feature	Description	N	%
Sociodemographic			
Gender (N = 275)	Female	138	50.2
	Male	137	49.8
	Missing	47	14.6
Age (N = 321)	≤44 Years	94	29.3
	45–54 Years	111	34.6
	≥55 Years	116	36.1
	Missing	1	0.3
Civil status (N = 321)	Single	40	12.9
	Married	216	69.7
	Other	65	17.4
	Missing	12	3.7
Academic qualifications (N = 264)	≤12 grade	44	16.7
	Graduate or Master	79	29.9
	PhD	141	53.4
	Missing	58	18.0
Organic units (N = 259)	Health sciences	17	6.6
	Natural and technical sciences	132	51.0
	Social and human Sciences	42	16.2
	Technical services	68	26.3
	Missing	63	19.6
Health self-rated			
BMI (kg/m^2^) (N = 304)	Normal	161	52.9
	Overweight	113	37.1
	Obese	30	9.9
	Missing	18	5.6
Self-health status (N = 312)	Excellent or very good	77	24.7
	Good	170	54.5
	Reasonable or weak	65	20.8
	Missing	10	3.1
Chronic pain (N = 301)	No	223	74.1
	Yes	78	25.9
	Missing	21	6.5
Physical habits			
Regular physical activity (N = 312)	No	182	58.3
	Yes	130	41.7
	Missing	10	3.1
Regular physical activity per week (N = 128)	<2 h/week	53	41.4
	2–3 h/week	34	26.6
	>3 h/week	41	32.0
	Missing	194	60.2
Soft mobility time spent, per day (N = 311)	<15 min/day	104	33.4
	15–30 min/day	112	36.0
	>30 min/day	95	30.5
	Missing	11	3.4
Eating habits			
Taking breakfast (N = 319)	All weekdays	290	90.9
	At least one day	27	8.4
	Never	2	0.6
	Missing	3	0.9
Eating fruits (N = 319)	All weekdays	226	70.8
	At least one day	91	28.5
	Never	2	0.6
	Missing	3	0.9
Eating vegetables (N = 307)	All weekdays	205	66.8
	At least one day	99	32.2
	Never	3	1.0
	Missing	5	1.6
Eating fast food (N = 319)	Never	128	40.1
	Once/week	152	47.6
	Twice or more/week	39	12.2
	Missing	3	0.9
Drinking soda (N = 318)	Never	157	49.4
	Once/week	103	32.4
	Twice or more/week	58	18.2
	Missing	4	1.2
Drinking alcohol/week (N = 320)	No	123	38.4
	Yes	197	61.6
	Missing	2	0.6

**Table 2:** Contingency table between CVDRisk1 and CVDRisk2.

	CVDRisk2			Statistical Results
CVDRisk1	No	Yes	Total	
No	56(19.2)	37(12.7)	93(31.8)	χ^2^(1) = 1.6; *p* = 0.203
Yes	104(35.6)	95(32.5)	199(68.2)	
Total	160(54.8)	132(45.2)	292	

Note. Percentages were calculated based on complete data for CVDRisk1 and CVDRisk2 (N = 292; corresponding to 90.7% of the collected sample).

**Table 3:** Associated factors for cardiovascular risk 1 (CVDRisk1) and risk 2 (CVDRisk2) from univariable and multivariable logistic regression.

	CVDRisk1						CVDRisk2					
	Univariable			Multivariable N = 228 (70.8%)			Univariable			Multivariable N = 262 (81.4%)		
Variables	N(%)	OR	95%IC	N(%)	OR	95%IC	N(%)	OR	95%IC	N(%)	OR	95%IC
Gender	n.s.											
Female	127(49.0)	1	-				131(49.8)	1		131(50.0)	1	
Male	132(51.0)	1.361	[0.803; 2.306]				132(50.2)	1.894	[1.157; 3.100]	131(50.0)	1.631	[0.974; 2.731]
Age	n.s.						n.s.					
≤44 Years	86(28.5)	1					89(28.8)	1				
45–54 Years	104(34.4)	1.268	[0.692; 2.325]				109(35.3)	1.180	[0.671; 2.074]			
≥55 Years	112(37.1)	1.541	[0.839; 2.827]				111(35.9)	1.061	[0.604; 1.862]			
Civil status	n.s.						n.s.					
Single	36(12.3)	1					39(13.0)	1				
Married	205(70.2)	0.927	[0.430; 1.996]				206(68.9)	1.266	[0.628; 2.553]			
Other	51(17.5)	1.163	[0.455; 2.973]				54(18.0)	1.723	[0.746; 3.981]			
Organic units							n.s.					
Health sciences	17(7.0)	1		17(7.4)	1		17(6.8)	1				
Natural and technical sciences	122(50.4)	2.306	[0.828; 6.425]	113(49.6)	1.950	[0.634; 6.002]	128(51.0)	1.042	[0.373; 2.913]			
Social and human sciences	38(15.7)	4.982	[1.418; 17.509]	35(15.4)	3.975	[0.984; 16.035]	41(16.3)	1.654	[0.527; 5.195]			
Technical services	65(26.9)	2.357	[0.796; 6.976]	63(27.6)	1.425	[0.430; 4.725]	65(25.9)	1.151	[0.390; 3.398]			
Body mass índex (kg/m^2^)	n.app.						n.s.					
Normal							155(52.7)	1				
Overweight							110(37.4)	1.335	[0.817; 2.183]			
Obese							29(9.9)	1.292	[0.583; 2.862]			
Self-health status							n.s.					
Excellent or very good	71(24.1)	0.609	[0.388; 1.230]	56(24.6)	0.849	[0.426; 1.694]	76(25.2)	0.939	[0.542; 1.627]			
Good	161(54.6)	1		129(56.6)	1		165(54.6)	1				
Reasonable or weak	63(21.4)	2.028	[0.996; 4.125]	43(18.9)	2.829	[1.063; 7.527]	61(20.1)	1.335	[0.741; 2.405]			
Regular physical exercise							n.app.					
No	172(58.3)	1.862	[1.131; 3.065]	129(55.6)	2.110	[1.131; 3.935]						
Yes	123(41.7)	1		99(43.4)	1							
Soft mobility time spent	n.s.						n.app.					
<15 min/day	96(32.8)	1.297	[0.702; 2.397]									
15–30 min/day	108(36.9)	1.333	[0.733; 2.426]									
>30 min/day	89(30.4)	1										
Taking breakfast	n.s.						n.s.					
All weekdays	272(90.4)	1					281(90.6)	1				
Other	29(9.6)	1.214	[0.517; 2.849]				29(9.3)	1.581	[0.733; 3.411]			
Eating fruits							n.app.					
All weekdays	211(70.0)	1		159(69.7)	1							
Other	90(29.9)	2.252	[1.251; 4.054]	69(30.3)	2.915	[1.426; 5.958]						
Drinking soda	n.s.											
Never	146(48.7)	1					153(49.5)	1		126(48.1)	1	-
Once/week	98(32.7)	0.965	[0.561; 1.660]				99(32.0)	2.213	[1.318; 3.715]	85(32.4)	2.400	[1.352; 4.262]
Twice or more/week	56(18.7)	1.620	[0.797; 3.295]				57(18.4)	3.429	[1.818; 6.467]	51(19.5)	3.678	[1.830; 7.392]
Drinking alcohol/week	n.s.						n.app.					
No	111(36.8)	1										
Yes	191(63.2)	1.003	[0.977; 1.030]									

Note. 95%CI: 95% confidence interval. n.app.: not applicable because this variable was used in the cardiovascular risk definition; n.s.: non-significant; CVDRisk1: cardiovascular risk 1 (at least one of these indicators: hypertension, diabetes, hypercholesterolaemia or obese (≥30 kg/m^2^)); CVDRisk2: cardiovascular risk 2 (at least one of these indicators: smoker, physical inactivity, unhealthy diet, or alcohol consumption (>25 gr/day)).

## Data Availability

The datasets that were analysed in this study are not publicly available but are available on request from the corresponding author.
